# The relationship between intolerance of uncertainty and academic burnout among university students: the mediating role of future time perspective and the moderating role of psychological flexibility

**DOI:** 10.3389/fpsyg.2026.1922858

**Published:** 2026-08-31

**Authors:** Zijie Wang, Youzeng Wang, Mengyi Pang, Zhenguo Xu

**Affiliations:** 1 School of Communication, Qufu Normal University, Rizhao, China; 2 Department of Basic Sciences, Shandong Water Conservancy Vocational College, Rizhao, China

**Keywords:** intolerance of uncertainty, academic burnout, future time perspective, psychological flexibility, moderated mediation

## Abstract

**Introduction:**

Academic burnout is an important manifestation of academic maladjustment among university students and is closely related to their academic development and mental health. The association between intolerance of uncertainty and academic burnout may partly reflect differences in students’ future-oriented cognition. This study examined the mediating role of future time perspective in the association between intolerance of uncertainty and academic burnout and tested the moderating role of psychological flexibility in the association between future time perspective and academic burnout.

**Methods:**

A convenience sample of 855 Chinese university students from five provinces participated in a cross-sectional questionnaire survey. Participants completed measures of intolerance of uncertainty, future time perspective, psychological flexibility, and academic burnout. Pearson correlations were calculated, and PROCESS Model 14 was estimated using 5,000 bootstrap resamples and HC3 robust standard errors. Analyses adjusted for sex, age, year of study, and academic discipline.

**Results:**

Higher intolerance of uncertainty was associated with higher academic burnout and lower future time perspective. Lower future time perspective was also associated with higher academic burnout. Future time perspective partially mediated the association between intolerance of uncertainty and academic burnout, and the direct effect of intolerance of uncertainty on academic burnout remained significant. Psychological flexibility moderated the future time perspective–academic burnout association, such that the negative association and the corresponding conditional indirect effect were stronger at higher levels of psychological flexibility. However, the interaction had a negligible incremental effect (ΔR^2^ = 0.007; Cohen’s f^2^ = 0.013).

**Discussion:**

This study found that intolerance of uncertainty was positively associated with academic burnout among university students and that future time perspective partially mediated this association. Psychological flexibility moderated the association between future time perspective and academic burnout, such that the negative association was stronger at higher levels of psychological flexibility. Because this study used a cross-sectional self-report design, the temporal ordering or causal relationships among the variables cannot be determined from these data. Future longitudinal research on academic burnout among university students is needed to verify these associations.

## 1 Introduction

### 1.1 Academic burnout among university students

Academic burnout is an important focus of research on higher education and student mental health. Burnout was originally conceptualized in occupational settings as a syndrome of exhaustion, detachment from work, and reduced efficacy (Maslach et al., 2001). Although university students are not employees in their academic role, they engage in sustained, formally evaluated academic work. This work includes coursework, attendance, assignments, examinations, achievement expectations, and preparation for postgraduate study or employment. Because these recurring demands are central to students’ academic role, they provide a clear conceptual basis for extending the burnout construct to higher education (Schaufeli et al., 2002). In this context, academic burnout refers to a study-related syndrome of exhaustion, cynicism toward study, and reduced academic efficacy. The Maslach Burnout Inventory–Student Survey (MBI-SS) operationalizes this role-based conception by directly assessing exhaustion, cynicism, and academic efficacy within students’ academic role; lower academic-efficacy scores indicate greater burnout (Schaufeli et al., 2002; Hu and Schaufeli, 2009).

Academic burnout is not short-term or occasional fatigue but a condition that persists in day-to-day academic activities (Maslach et al., 2001; Schaufeli et al., 2002). It differs from temporary fatigue during examinations; its influence is not confined to a single examination but is reflected across multiple aspects of students’ everyday learning. Students experiencing burnout may feel depleted by academic work, become detached from learning, and lose confidence in their academic competence. Burnout has been documented among university students across 24 low- and middle-income countries (Kaggwa et al., 2021). Across school, college, and university samples, academic burnout was negatively associated with academic achievement (Madigan and Curran, 2021). Perceived life stress across several domains has also been associated with academic burnout among college students (Lin and Huang, 2014). Together, these findings position academic burnout as a consequential and persistent condition related to both academic functioning and students’ broader stress experiences.

Prior research has examined academic stress (Lin and Huang, 2014; Pascoe et al., 2020) and academic motivation or engagement (Howard et al., 2021; Salmela-Aro and Read, 2017). It has also examined academic self-efficacy (Bresó et al., 2011), emotion regulation (Iuga and David, 2024), and perfectionistic tendencies (Zhang et al., 2007; Hill and Curran, 2016). University students also navigate uncertainty about examination results, academic performance, postgraduate study, career choice, employment, and broader future development. These experiences combine demanding tasks with outcomes that can be predicted or controlled only to a limited extent. Students differ in how they appraise and respond to such uncertainty. Against existing explanations of academic burnout based mainly on workload, motivation, and general stress, incorporating intolerance of uncertainty makes it possible to further examine how students view and respond to academic and developmental prospects that have not yet been determined.

### 1.2 Intolerance of uncertainty and academic burnout

Intolerance of uncertainty (IU) refers to a tendency to respond negatively at cognitive, emotional, and behavioral levels when facing uncertain or unpredictable situations (Freeston et al., 1994; Buhr and Dugas, 2002). Individuals with higher intolerance of uncertainty are more likely to experience worry and distress when outcomes are unclear. They are also more likely to overestimate the likelihood of negative events, seek excessive certainty, or avoid situations that cannot be fully predicted or controlled (Dugas et al., 1998; Carleton et al., 2007). These tendencies are especially relevant to higher education, where many important academic and career outcomes are delayed, not fully determined, and only partly under personal control.

A first theoretical route from IU to academic burnout involves threat appraisal and perceived control. University students frequently encounter uncertainty about grades, opportunities for postgraduate study, and employment prospects. Students with higher IU may be more likely to view this uncertainty as a sign of danger or failure rather than as a manageable challenge (Grupe and Nitschke, 2013; Carleton, 2016). Control-value theory holds that perceived control over achievement situations shapes academic emotions, motivation, and engagement (Pekrun, 2006). Thus, the tendency to view uncertainty as a threat may be associated with lower perceived control, more worry, stronger negative achievement emotions, and greater reliance on avoidance strategies. Certainty-seeking and avoidance may provide short-term relief, yet repeated withdrawal can interfere with preparation, help-seeking, and sustained engagement. Academic demands that are persistently appraised as uncontrollable may therefore be associated with greater exhaustion, cynicism toward study, and reduced academic efficacy.

A second theoretical route concerns the sustained expenditure of cognitive and emotional resources. Conservation of resources theory proposes that stress arises when valued resources are threatened, lost, or heavily invested without adequate return (Hobfoll, 1989; Hobfoll et al., 2018). Students with higher IU may devote sustained attention and emotional effort to predicting, monitoring, and controlling unclear outcomes. Persistent worry can occupy working-memory resources and weaken goal-directed attentional control (Eysenck et al., 2007; Hirsch and Mathews, 2012; Moran, 2016). Students may then have fewer resources for studying, recovery, and adaptive engagement, making exhaustion and detachment more likely. Among Chinese university students, Qiang et al. (2024) reported an indirect association between IU and academic burnout through self-regulatory fatigue. This finding is consistent with a resource pathway in which managing uncertainty may consume self-regulatory capacity and leave fewer resources for sustained academic functioning. Together, threat-focused appraisal and sustained resource expenditure provide complementary theoretical reasons why higher IU should be associated with greater academic burnout.

In summary, viewing uncertainty as a threat and continuously expending cognitive and emotional resources to cope with uncertainty provide a theoretical basis for the positive association between IU and academic burnout. Accordingly, the present study proposed the following hypothesis:

*H1*: Among university students, intolerance of uncertainty is positively associated with academic burnout.

### 1.3 The mediating role of future time perspective

Future time perspective (FTP) refers to how individuals understand future possibilities and goals and connect present behavior with long-term outcomes (Zimbardo and Boyd, 1999; Seginer, 2003). It involves not only positive, negative, or confused views of the future, but also whether future goals are clear, whether individuals can plan for them, and whether they can sustain effort toward those goals (Lyu and Huang, 2016). FTP is therefore not simply optimism about the future; it reflects whether individuals can take a longer-term view of their future, establish goals, and organize current action accordingly. For university students, FTP concerns whether they can connect current learning with long-term goals such as postgraduate study, career development, and personal growth, thereby understanding the meaning of present learning (Husman and Shell, 2008).

The future is inherently uncertain, making it a particularly relevant domain for IU. Students with higher IU may allocate disproportionate attention to possible failure, uncontrollable outcomes, and barriers to future development. This threat-focused orientation may be associated with more negative or confused evaluations of the future, as well as less clarity and planning. That pattern maps directly onto the content domains assessed by the FTPS-AYA (Lyu and Huang, 2016). Studies using the FTPS-AYA have reported associations of its dimensions with self-esteem and self-control (Lyu et al., 2019; Hao et al., 2024). These associations support the scale’s relevance to the personal resources involved in organizing and regulating future-directed action. Taken together, IU theory and the FTPS-AYA evidence support the expectation that higher IU will be associated with lower FTP.

For university students, higher FTP helps them understand the value of current learning tasks from a long-term developmental perspective and connect short-term academic effort with future goals, thereby strengthening perceived meaning in learning, goal clarity, and sustained engagement (Seginer, 2003; Husman and Shell, 2008). When students can place temporary difficulties within a broader framework of future development, they are more likely to maintain goal-directed engagement and use planning, persistence, and goal-management strategies to meet academic challenges (Simons et al., 2004; De Bilde et al., 2011). Higher FTP may therefore be associated with less emotional exhaustion and detachment from learning, together with stronger academic efficacy. In contrast, students with lower FTP may struggle to recognize the long-term value of current learning tasks, which may weaken academic engagement and goal persistence and make experiences of meaninglessness, low efficacy, and burnout more likely (Zimbardo and Boyd, 1999; Simons et al., 2004). Previous research has also shown a negative association between future time perspective and academic burnout among university students (Hong and Hanafi, 2024).

In summary, students with higher IU may find it more difficult to develop a clear, positive, and controllable view of the future, whereas lower FTP may make it more difficult to understand the meaning of current learning and sustain engagement, thereby increasing the risk of academic burnout. FTP may therefore mediate the association between IU and academic burnout. Accordingly, the present study proposed the following hypothesis:

*H2*: Future time perspective mediates the association between intolerance of uncertainty and academic burnout.

### 1.4 The moderating role of psychological flexibility

The association between FTP and academic burnout may depend on psychological flexibility. Psychological flexibility refers to the capacity to remain open and aware when difficult thoughts, emotions, or uncertain experiences are present. It also entails acting in accordance with personal values and goals despite those experiences (Hayes et al., 2006; Kashdan and Rottenberg, 2010). Its defining feature is the capacity to maintain value-consistent action while difficult internal experiences remain present. In academic contexts, this capacity may be relevant when students must continue goal-directed activity despite anxiety, frustration, or uncertainty.

FTP and psychological flexibility are proposed as complementary rather than interchangeable resources. The resource-caravan principle holds that resources tend to aggregate and operate together (Hobfoll et al., 2018). Applied to university study, this principle suggests that future direction and goal-directed self-regulation should operate jointly. FTP provides students with temporal direction, goal clarity, and perceived instrumentality. Psychological flexibility enables students to act on that direction while distress remains present. At higher levels of psychological flexibility, students should be better able to translate future goals into planned academic activity despite anxiety or frustration. At lower levels, experiential avoidance may more readily interrupt this translation from future direction to academic action. Accordingly, psychological flexibility was expected to be more directly relevant to the FTP–academic burnout association than to the IU–FTP association. The former concerns whether future direction remains connected with goal-consistent action under distress, whereas the latter concerns how uncertainty appraisal is associated with the clarity and organization of future-oriented cognition. The negative FTP–burnout association is therefore expected to be stronger when psychological flexibility is higher.

This moderating role can be further understood from the premise that future goals do not automatically translate into actual academic action. Future time perspective helps students form long-term goals and recognize the value of current learning, but when anxiety, fear of failure, or frustration remains present, students must continue to act in accordance with their values and goals for those goals to keep guiding behavior (Hayes et al., 2006; Kashdan and Rottenberg, 2010). Research with university students has shown that psychological flexibility is closely related to time and effort management and is independently associated with academic procrastination; acceptance-based behavioral intervention has also been more effective among students who place greater value on academics (Glick and Orsillo, 2015; Hailikari et al., 2021). Valuing academic goals therefore does not necessarily ensure continued academic engagement; whether students can keep acting in the face of anxiety and frustration may be an important condition for putting those goals into practice. Psychological flexibility has also been negatively associated with study-related burnout among university students, and research in occupational settings has found that work-related psychological flexibility moderates the association between exhaustion and disengagement from work (Asikainen and Katajavuori, 2023; Ruiz and Odriozola-González, 2017). Taken together, psychological flexibility may be relevant to whether future goals continue to be translated into academic action under pressure, providing a theoretical basis for treating it as a moderator of the association between future time perspective and academic burnout.

Previous research has linked psychological flexibility to better mental health and adaptation under stress (Kashdan and Rottenberg, 2010; Gloster et al., 2017). Together with the preceding account of how multiple psychological resources may operate jointly, the present study examined psychological flexibility as a moderator of the association between future time perspective and academic burnout. Accordingly, the present study proposed the following hypothesis:

*H3*: Psychological flexibility moderates the association between future time perspective and academic burnout.

### 1.5 Hypotheses and moderated mediation model of this study

**Figure 1:**
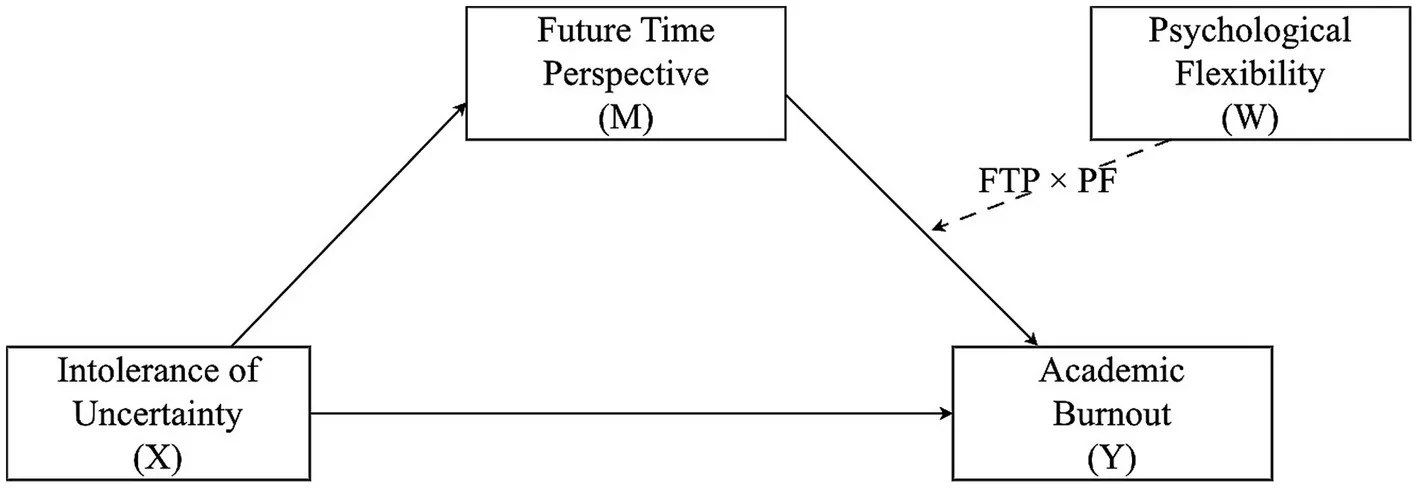
Conceptual model of the hypothesized associations. Solid arrows denote the direct paths; the sequence from intolerance of uncertainty to future time perspective to academic burnout constitutes the indirect path. The dashed arrow denotes moderation of the future time perspective–academic burnout association by psychological flexibility.

The present study examined these associations in a sample of Chinese university students. At the theoretical level, it integrated uncertainty appraisal, future orientation, and self-regulatory action within a single conditional process model. The hypothesized moderated mediation model is shown in Figure 1. Intolerance of uncertainty was the predictor, academic burnout was the outcome, future time perspective was the mediator, and psychological flexibility was the moderator. Specifically, psychological flexibility was hypothesized to moderate the second stage of the mediating pathway, namely, the association between future time perspective and academic burnout. This structure corresponds to PROCESS Model 14 (Hayes, 2022).

The study hypothesized that higher intolerance of uncertainty would be associated with lower future time perspective, which would in turn be associated with higher academic burnout, and that psychological flexibility would moderate the association between future time perspective and academic burnout. Accordingly, in addition to testing the mediating role of future time perspective and the moderating role of psychological flexibility, the study examined whether the indirect association between intolerance of uncertainty and academic burnout through future time perspective varied across levels of psychological flexibility.

## 2 Materials and methods

### 2.1 Participants

This cross-sectional questionnaire study examined the associations among intolerance of uncertainty, future time perspective, psychological flexibility, and academic burnout and estimated a moderated mediation model. Convenience sampling was used to recruit students from universities in Shandong, Henan, Jiangsu, Hebei, and Anhui provinces. To ensure sample appropriateness and data validity, clear inclusion and exclusion criteria were established before data collection. The inclusion criteria were as follows: first, participants were currently enrolled university students; second, they were able to understand the questionnaire items and complete the questionnaire independently; third, they voluntarily participated in the study and provided informed consent. Exclusion criteria included excessive missing questionnaire data, an excessively short response time, obvious patterned responses, repeated submissions, or logical inconsistencies between responses. A total of 948 questionnaires were distributed, and 889 were returned, yielding a response rate of 93.8%. After 34 invalid questionnaires were excluded according to the above criteria, 855 valid questionnaires remained, accounting for 96.2% of the returned questionnaires and 90.2% of all distributed questionnaires.

Participants were aged 18–24 years, with a mean age of 19.64 years (*SD* = 0.93). Among them, 363 were male students (42.5%) and 492 were female students (57.5%). In terms of year of study, 331 were first-year students (38.7%), 226 were second-year students (26.4%), 133 were third-year students (15.6%), and 165 were students in their fourth year or later (19.3%). In terms of academic discipline, participants represented literature, science, education, economics, engineering, law, philosophy, agriculture, and arts. Engineering students constituted the largest group, with 476 participants (55.7%), followed by agriculture students, with 183 participants (21.4%), and science students, with 168 participants (19.6%). The remaining 28 participants (3.3%) were enrolled in literature, education, economics, law, philosophy, or arts.

### 2.2 Research procedure

This study followed ethical principles for psychological research and obtained approval from the Ethics Committee of Qufu Normal University before data collection (approval number: 2026135). Before the study was implemented, the researchers obtained permission from the relevant school administrative departments. Before completing the questionnaire, all participants were informed of the study purpose, the voluntary nature of participation, the anonymity of their responses, and the principles of data confidentiality. Participants were also informed that the questionnaire data would be used only for academic research and would not affect their course grades, academic evaluations, or rights and welfare. All participants took part in the study after providing informed consent.

Questionnaires were administered through a combination of online and offline methods. Online responses were collected via Wenjuanxing, and the first page explained the study purpose, response requirements, confidentiality principles, and relevant instructions. Participants completed the questionnaire independently based on their own experiences. Paper questionnaires were administered by trained researchers. Before completing the questionnaires, participants were informed that there were no right or wrong answers and were asked to respond honestly. Paper questionnaires were completed independently by participants and collected on site after completion.

After the survey, all questionnaires were screened for completeness and response quality. Questionnaires that were incomplete, had excessive missing values, had an excessively short response time, were repeated submissions, or showed obvious patterned responses were excluded. The final valid questionnaires were coded and checked before analysis. All data were processed anonymously, and the final data were used only for the statistical analyses of this study.

### 2.3 Measures

#### 2.3.1 Intolerance of uncertainty

The Intolerance of Uncertainty Scale–Short Form (IUS-12) was used to assess intolerance of uncertainty among university students. The IUS-12 was developed by Carleton et al. (2007) as a shortened version of the original 27-item IUS and was later translated and evaluated among Chinese university students by Zhang et al. (2017). The Chinese IUS-12 contains 12 items and is mainly used to measure cognitive, emotional, and behavioral responses to uncertain, ambiguous, or unpredictable situations. Items are rated on a 5-point Likert scale ranging from 1 = “not at all characteristic of me” to 5 = “entirely characteristic of me.” Higher scores indicate a higher level of intolerance of uncertainty.

Zhang et al. (2017) reported that the Chinese IUS-12 comprised two dimensions, prospective anxiety and inhibitory anxiety. In their Chinese university student sample, the KMO value was 0.833 and Bartlett’s test of sphericity was statistically significant (*p* < 0.001). As reported by Zhang et al. (2017), confirmatory factor analysis produced CFI = 0.926, TLI = 0.908, SRMR = 0.057, and RMSEA = 0.001, supporting the reported two-factor structure.

In the present study, the mean item score across the 12 items was used as the intolerance of uncertainty composite score. The overall Cronbach’s *α* coefficient was 0.909; the coefficients for prospective anxiety and inhibitory anxiety were 0.884 and 0.831, respectively, indicating good internal consistency.

#### 2.3.2 Academic burnout

Academic burnout was measured using the Maslach Burnout Inventory–Student Survey (MBI-SS). This scale assesses students’ burnout experiences in learning activities and has been shown to be applicable to Chinese student populations (Schaufeli et al., 2002; Hu and Schaufeli, 2009). The MBI-SS contains 15 items and consists of three dimensions: emotional exhaustion, cynicism, and academic efficacy. Emotional exhaustion refers to students’ feelings of fatigue and resource depletion caused by academic demands; cynicism refers to students’ detached, negative, or skeptical attitudes toward learning activities; and academic efficacy reflects students’ perceived academic competence and effectiveness.

Because academic efficacy is a positive dimension, its items were reverse-scored when calculating the academic burnout composite mean score and were therefore interpreted as reduced academic efficacy (Chae et al., 2020; Calcatin et al., 2022; Zhu et al., 2023). The scale uses a 7-point frequency response format. According to the questionnaire setting in this study, item scores ranged from 0 = “never” to 6 = “every day.” Items in the academic efficacy dimension were reverse-scored according to the 0–6 scoring rule and then averaged with the emotional exhaustion and cynicism items. Higher composite mean scores indicate higher levels of academic burnout.

In this study, the overall Cronbach’s α coefficient for the academic burnout scale was 0.918; the coefficients for emotional exhaustion, cynicism, and low academic efficacy were 0.891, 0.894, and 0.877, respectively. The KMO value was 0.939, and Bartlett’s test of sphericity was significant, χ^2^(105) = 7,772.644, *p* < 0.001, indicating that the data were suitable for factor analysis. Confirmatory factor analysis yielded χ^2^/*df* = 3.578, RMSEA = 0.075, GFI = 0.898, NFI = 0.956, IFI = 0.970, and CFI = 0.970, indicating good reliability and structural validity of the scale in the present sample.

#### 2.3.3 Future time perspective

Future time perspective was measured using the Future Time Perspective Scale for Adolescents and Young Adults (FTPS-AYA). The scale was developed by Lyu and Huang (2016) to assess adolescents’ and young adults’ cognitive, emotional, and behavioral tendencies regarding the future. The scale contains 28 items and consists of six dimensions: future-negative, future-positive, future-confusion, future-perseverant, future-perspicuity, and future-planning. The future-negative dimension reflects individuals’ negative emotions and pessimistic expectations about the future; future-positive reflects positive emotions and expectations regarding the future; future-confusion reflects uncertainty and ambiguity about the future; future-perseverant reflects persistence in pursuing future goals; future-perspicuity reflects a sense of clarity and control regarding the future; and future-planning reflects the tendency to plan and organize future tasks.

The scale uses a 5-point Likert response format ranging from 1 = “strongly disagree” to 5 = “strongly agree.” When calculating the composite mean score, items in the future-negative and future-confusion dimensions, together with the designated reverse-scored items in the future-planning dimension, were reverse-scored. After reverse scoring, higher composite mean scores indicate a more positive, clearer, and more goal-oriented orientation toward the future.

In this study, the overall Cronbach’s *α* coefficient was 0.954; the coefficients for future-negative, future-positive, future-confusion, future-perseverant, future-perspicuity, and future-planning were 0.897, 0.895, 0.837, 0.888, 0.845, and 0.841, respectively. The KMO value was 0.969, and Bartlett’s test of sphericity was statistically significant, χ^2^(378) = 15,384.235, *p* < 0.001, indicating that the item-correlation matrix was suitable for factor analysis. Confirmatory factor analysis of the hypothesized six correlated first-order factor structure showed χ^2^/*df* = 3.377, RMSEA = 0.053, 90% CI [0.049, 0.056], IFI = 0.981, CFI = 0.981, TLI = 0.979, and SRMR = 0.034, supporting the factorial validity of this structure.

#### 2.3.4 Psychological flexibility

Psychological flexibility was measured using the psychological flexibility subscale of the Multidimensional Psychological Flexibility Inventory-24 (MPFI-24). The MPFI-24 was developed from the original Multidimensional Psychological Flexibility Inventory, and its Chinese short form has shown acceptable reliability and validity in Chinese samples (Rolffs et al., 2018; Fang et al., 2024). The inventory includes psychological flexibility and psychological inflexibility subscales. The psychological flexibility subscale comprises acceptance, present-moment awareness, self-as-context, defusion, values, and committed action. The psychological inflexibility subscale comprises experiential avoidance, lack of contact with the present moment, self-as-content, fusion, lack of contact with values, and inaction.

Although psychological flexibility and psychological inflexibility are related, they cannot simply be understood as opposite levels of a single dimension; rather, they represent related but distinct adaptive and maladaptive psychological regulation processes (Rolffs et al., 2018; Azadfar et al., 2022). Directly reverse-scoring psychological inflexibility items and combining them with psychological flexibility items could therefore confound two distinct processes. Because the present study focused on psychological flexibility rather than psychological inflexibility, and because the MPFI-24 permits separate composite scores for the two constructs, only the 12-item psychological flexibility subscale was used in the analyses (Rolffs et al., 2018; Fang et al., 2024).

Items are rated on a 6-point response scale ranging from 1 = “never true” to 6 = “always true.” The mean of the 12 item scores was used as the psychological flexibility score, with higher scores indicating greater psychological flexibility. In this study, the Cronbach’s *α* coefficient of the psychological flexibility subscale was 0.951, indicating good internal consistency. The KMO value was 0.915, and Bartlett’s test of sphericity was statistically significant (*p* < 0.001), indicating that the data were suitable for factor analysis. Confirmatory factor analysis of the specified model with six correlated first-order factors yielded χ^2^/*df* = 1.731, RMSEA = 0.029, GFI = 0.959, NFI = 0.978, IFI = 0.991, and CFI = 0.991, indicating acceptable model fit.

### 2.4 Data analysis

This study used IBM SPSS Statistics 31, PROCESS 5.0, R 4.6.1, lavaan 0.6–21, and semTools 0.5–9 for data processing and statistical analysis. SPSS and PROCESS were used for analyses based on the scale composite scores, whereas R, lavaan, and semTools were used for confirmatory factor analyses, measurement-model comparisons, HTMT₂ calculations, and latent-variable analyses. First, invalid questionnaires were excluded according to the above screening criteria, and reverse-keyed items were recoded according to the scoring instructions. Mean scale scores and dimension scores were then calculated for intolerance of uncertainty, academic burnout, future time perspective, and psychological flexibility in accordance with the scoring rules for each scale. Cronbach’s α coefficients were used to assess internal consistency; KMO tests and Bartlett’s tests of sphericity were used to assess the factorability of the item-correlation matrices; and confirmatory factor analysis was used to evaluate the fit of the specified factor models. Harman’s single-factor test provided a preliminary assessment of potential common method variance (CMV) (Podsakoff et al., 2003). Descriptive statistics and Pearson correlation analyses were then conducted to examine the basic characteristics of the main variables and their correlations. Variance inflation factors (VIFs) were calculated to assess multicollinearity.

Finally, the PROCESS macro in SPSS was used to test the moderated mediation model (Hayes, 2022). Specifically, PROCESS Model 14 was estimated with intolerance of uncertainty as the independent variable, academic burnout as the dependent variable, future time perspective as the mediator, and psychological flexibility as the moderator, thereby testing whether psychological flexibility moderated the association between future time perspective and academic burnout. A nonparametric bootstrap procedure with 5,000 resamples was used to estimate the indirect effect, the conditional indirect effects at different levels of psychological flexibility, and the 95% confidence interval for the index of moderated mediation. An effect was considered statistically significant when its 95% confidence interval did not include 0. HC3 robust standard errors were used to provide heteroskedasticity-consistent inference for the regression coefficients.

The study first conducted PROCESS Model 14 using the composite score for each scale and then performed supplementary checks of the measurement structure, latent-variable interaction, and alternative moderation placements to provide complementary evidence concerning the focal model. The FTPS-AYA was evaluated using a bifactor model, and the four focal constructs were examined in a joint higher-order measurement model estimated with WLSMV. Discriminant validity was additionally assessed using HTMT₂ computed from absolute indicator correlations with semTools:htmt() (htmt2 = TRUE; Roemer et al., 2021). An MLR product-indicator latent interaction model was then fitted using lavaan (version 0.6–21) with the same structural paths and covariate specification as the primary observed-score PROCESS Model 14. Sex, age, year of study, and academic discipline were included as covariates in both the FTP and academic-burnout equations. PROCESS Models 7 and 5 were estimated separately to examine alternative placements of the moderation effect. These were supplementary checks; PROCESS Model 14 based on the scale composite scores remained the primary analysis.

## 3 Results

### 3.1 Common method variance assessment and multicollinearity diagnostics

Because the main variables in this study were collected using self-report questionnaires, Harman’s single-factor test was used as a preliminary assessment of potential CMV. The results showed that 10 factors had eigenvalues greater than 1 and that the first unrotated factor explained 30.211% of the variance, below the 40% reference criterion commonly used in the relevant methodological literature (Tang and Wen, 2020). Following the basic logic of Harman’s single-factor test, neither a single factor was extracted nor did the first factor explain most of the covariance among the questionnaire items, providing no evidence that a single factor dominated the item covariance structure (Podsakoff et al., 2003).

To supplement Harman’s single-factor test, a 67-item single-factor CFA was compared with a measurement model containing four higher-order factors using WLSMV estimation. The single-factor model showed substantially poorer fit (scaled χ^2^/*df* = 9.200, CFI = 0.755, TLI = 0.747, RMSEA = 0.098, 90% CI [0.097, 0.099], SRMR = 0.125) than the four higher-order-factor measurement model (scaled χ^2^/*df* = 2.635, CFI = 0.951, TLI = 0.950, RMSEA = 0.044, 90% CI [0.042, 0.045], SRMR = 0.063). The better fit of the four higher-order-factor model indicated that the item covariance was represented more adequately by the four theoretical constructs than by a single factor. Because this comparison did not include a method factor in addition to the four theoretical factors, it should not be interpreted as a direct estimate of CMV. Taken together, Harman’s single-factor test and the single-factor CFA comparison did not support a covariance structure dominated by one factor; the clear advantage of the four higher-order-factor model also provided a preliminary measurement-level basis for including the four focal constructs in the moderated mediation analysis.

Before testing the moderated mediation model, multicollinearity diagnostics were conducted. In the model with academic burnout as the dependent variable, the VIF values for intolerance of uncertainty, future time perspective, psychological flexibility, and their interaction term were 1.328, 1.748, 1.413, and 1.135, respectively. All values were below 5.00, indicating no serious multicollinearity among these focal predictors (Kim, 2019).

### 3.2 Descriptive statistics and correlation analysis

Table 1 presents the means, standard deviations, and correlation coefficients of the main study variables. The mean scores for academic burnout, intolerance of uncertainty, future time perspective, and psychological flexibility were 2.143, 2.705, 3.602, and 4.149, respectively. The corresponding standard deviations were 1.212, 0.816, 0.719, and 1.021.

**Table 1:** Means, standard deviations, and correlation coefficients of study variables.

Variable	*M*	*SD*	1	2	3	4
1. Intolerance of uncertainty	2.705	0.816	1			
2. Future time perspective	3.602	0.719	−0.410***	1		
3. Psychological flexibility	4.149	1.021	−0.081*	0.485***	1	
4. Academic burnout	2.143	1.212	0.411***	−0.638***	−0.376***	1

*N* = 855. Values are Pearson correlation coefficients. All p values are two-tailed and were not adjusted for multiple comparisons. *p < 0.05, **p < 0.01, ***p < 0.001.

Correlation analysis showed that intolerance of uncertainty was positively correlated with academic burnout, indicating that higher intolerance of uncertainty was associated with higher academic burnout (*r* = 0.411, *p* < 0.001). Intolerance of uncertainty was negatively correlated with future time perspective, indicating that higher intolerance of uncertainty was associated with lower future time perspective (*r* = −0.410, *p* < 0.001). Intolerance of uncertainty was also weakly and negatively correlated with psychological flexibility (*r* = −0.081, *p* = 0.018).

In addition, psychological flexibility was positively correlated with future time perspective, indicating that higher psychological flexibility was associated with higher future time perspective (*r* = 0.485, *p* < 0.001). Future time perspective was negatively correlated with academic burnout, indicating that higher future time perspective was associated with lower academic burnout (*r* = −0.638, *p* < 0.001). Psychological flexibility was negatively correlated with academic burnout, indicating that higher psychological flexibility was associated with lower academic burnout (*r* = −0.376, *p* < 0.001). Overall, the directions of the correlations among the main variables were generally consistent with the theoretical expectations of this study, providing preliminary support for the subsequent mediation and moderation analyses.

### 3.3 Direct association between intolerance of uncertainty and academic burnout

The zero-order correlation between intolerance of uncertainty and academic burnout was positive and significant (*r* = 0.411, *p* < 0.001), supporting H1. In the full PROCESS Model 14, intolerance of uncertainty also retained a significant positive direct effect on academic burnout after future time perspective, psychological flexibility, their interaction, and the covariates were included (*B* = 0.286, HC3 *SE* = 0.050, *t* = 5.717, *p* < 0.001, 95% CI [0.188, 0.384]).

### 3.4 Mediating effect of future time perspective

The mediating role of future time perspective between intolerance of uncertainty and academic burnout was then tested. The results showed that intolerance of uncertainty was significantly and negatively associated with future time perspective (*B* = −0.364, HC3 *SE* = 0.029, *t* = −12.725, *p* < 0.001, 95% CI [−0.420, −0.308]), indicating that higher intolerance of uncertainty was associated with lower future time perspective.

Further analysis using academic burnout as the dependent variable showed that, at the mean level of psychological flexibility, future time perspective was significantly and negatively associated with academic burnout (*B* = −0.791, HC3 *SE* = 0.060, *t* = −13.219, *p* < 0.001, 95% CI [−0.909, −0.674]). At this level of psychological flexibility, the conditional indirect effect of intolerance of uncertainty on academic burnout through future time perspective was 0.288 (Boot*SE* = 0.033, 95% CI [0.226, 0.356]). Because the bootstrap confidence interval did not include 0, the indirect effect was significant and H2 was supported. The direct effect of intolerance of uncertainty on academic burnout also remained significant after future time perspective, psychological flexibility, and their interaction term were included (*B* = 0.286, HC3 *SE* = 0.050, *t* = 5.717, *p* < 0.001). The indirect effect was statistically significant, while the direct effect remained statistically significant. The regression path coefficients are shown in Table 2, and the conditional indirect effects are shown in Table 3.

**Table 2:** Results of the moderated mediation analysis (PROCESS model 14).

Outcome variable	Predictor variable	*B*	HC3 *SE*	*t*	*p*	95% CI
Future time perspective	Intolerance of uncertainty	−0.364	0.029	−12.725	< 0.001	[−0.420, −0.308]
Academic burnout	Intolerance of uncertainty	0.286	0.050	5.717	< 0.001	[0.188, 0.384]
Academic burnout	Future time perspective	−0.791	0.060	−13.219	< 0.001	[−0.909, −0.674]
Academic burnout	Psychological flexibility	−0.146	0.037	−3.963	< 0.001	[−0.218, −0.074]
Academic burnout	Future time perspective × psychological flexibility	−0.141	0.039	−3.571	< 0.001	[−0.218, −0.063]

*N* = 855. *B* denotes an unstandardized coefficient, and HC3 SE denotes a heteroskedasticity-consistent robust standard error. Future time perspective and psychological flexibility were mean-centered. Models adjusted for the prespecified covariates. In the academic-burnout equation, lower-order coefficients are evaluated at the other interacting variable’s sample mean. Residual dfs were 845 for the future-time-perspective equation and 842 for the academic-burnout equation.

**Table 3:** Conditional indirect effects at different levels of psychological flexibility.

Conditional indirect effect	Effect value	Boot*SE*	BootLLCI	BootULCI
Low psychological flexibility	0.236	0.036	0.169	0.309
Mean psychological flexibility	0.288	0.033	0.226	0.356
High psychological flexibility	0.340	0.036	0.273	0.416
Index of moderated mediation	0.051	0.015	0.023	0.082

*N* = 855. Percentile-bootstrap CIs were based on 5,000 resamples. Low, mean, and high psychological flexibility correspond to −1 SD, the sample mean, and +1 SD, respectively. Effects and the index of moderated mediation are unstandardized.

### 3.5 Moderating effect of psychological flexibility

**Figure 2:**
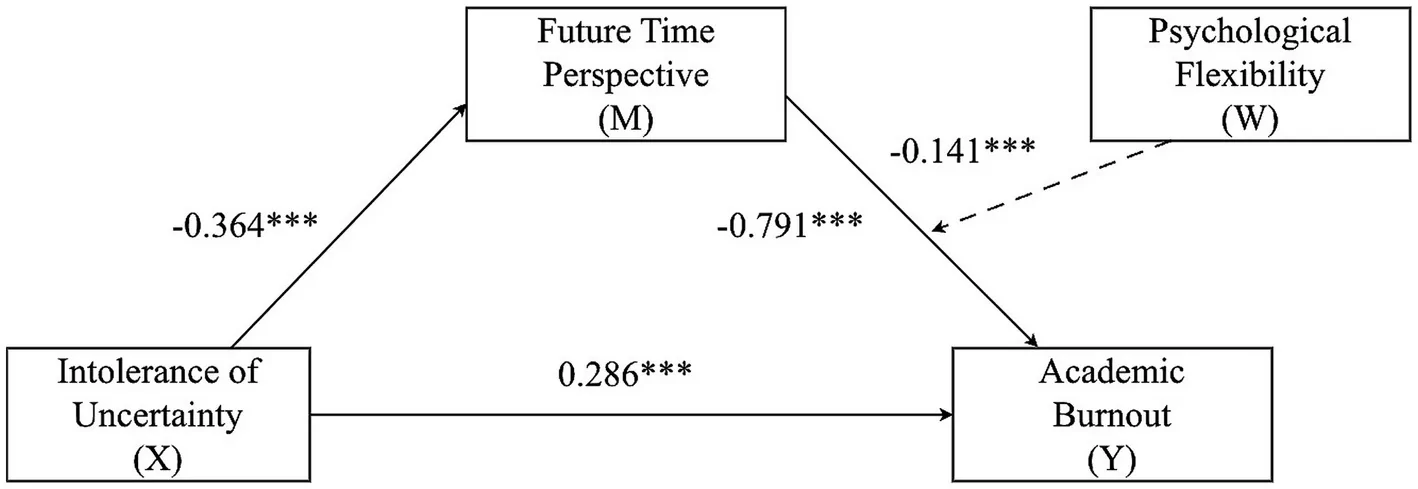
Path coefficient diagram of the moderated mediation model. *N* = 855. Values shown are selected focal unstandardized coefficients (*B*); ***p < 0.001. Psychological flexibility is shown as moderating the FTP–academic burnout path by a dashed arrow; *B* = −0.141*** denotes the FTP × PF interaction. The psychological flexibility–academic burnout main effect (*B* = −0.146, *p* < 0.001) and covariate paths are omitted for clarity. Complete focal regression coefficients are reported in Table 2, and full covariate estimates are reported in Table 4. Lower-order effects are evaluated when the other interacting variable is at its sample mean.

This study first tested whether psychological flexibility moderated the association between future time perspective and academic burnout. It then examined whether the indirect effect of intolerance of uncertainty on academic burnout through future time perspective varied across levels of psychological flexibility. In this model, psychological flexibility was specified as the moderator of the association between future time perspective and academic burnout. The regression path coefficients for the moderated mediation model are presented in Table 2, the path diagram with selected focal unstandardized regression coefficients is shown in Figure 2, the hierarchical regression results are shown in Table 4, and the conditional indirect effects are shown in Table 3.

**Table 4:** Hierarchical regression analysis of academic burnout.

Panel A. Step 1: covariates						
Predictor	*B*	β	HC3 *SE*	*t*(846)	*p*	95% CI
Female vs. male	0.067	0.028	0.085	0.795	0.427	[−0.099, 0.234]
Age	−0.177	−0.136	0.047	−3.796	< 0.001	[−0.269, −0.086]
Second year vs. first year	−0.111	−0.040	0.121	−0.915	0.360	[−0.348, 0.127]
Third year vs. first year	−0.465	−0.139	0.123	−3.765	< 0.001	[−0.707, −0.223]
Fourth year vs. first year	−0.104	−0.034	0.124	−0.841	0.400	[−0.348, 0.139]
Science vs. engineering	−0.243	−0.080	0.109	−2.232	0.026	[−0.456, −0.029]
Agriculture vs. engineering	−0.151	−0.051	0.110	−1.379	0.168	[−0.366, 0.064]
Other discipline vs. engineering	−0.767	−0.113	0.197	−3.888	< 0.001	[−1.154, −0.380]

Panel B. Step 2: covariates and focal predictors						
Predictor	*B*	β	HC3 *SE*	*t*(843)	*p*	95% CI
Female vs. male	0.051	0.021	0.068	0.746	0.456	[−0.083, 0.185]
Age	−0.037	−0.029	0.039	−0.947	0.344	[−0.114, 0.040]
Second year vs. first year	−0.023	−0.008	0.094	−0.244	0.808	[−0.207, 0.161]
Third year vs. first year	−0.343	−0.103	0.078	−4.380	< 0.001	[−0.497, −0.189]
Fourth year vs. first year	−0.013	−0.004	0.093	−0.140	0.888	[−0.195, 0.169]
Science vs. engineering	−0.271	−0.089	0.089	−3.044	0.002	[−0.445, −0.096]
Agriculture vs. engineering	0.041	0.014	0.084	0.489	0.625	[−0.124, 0.205]
Other discipline vs. engineering	−0.816	−0.120	0.120	−6.797	< 0.001	[−1.051, −0.580]
Intolerance of uncertainty	0.288	0.194	0.050	5.717	< 0.001	[0.189, 0.387]
Future time perspective	−0.826	−0.490	0.059	−13.878	< 0.001	[−0.942, −0.709]
Psychological flexibility	−0.160	−0.135	0.038	−4.243	< 0.001	[−0.234, −0.086]

Panel C. Step 3: Covariates, focal predictors, and interaction						
Predictor	*B*	β	HC3 *SE*	*t*(842)	*p*	95% CI
Female vs. male	0.060	0.024	0.068	0.883	0.378	[−0.073, 0.193]
Age	−0.041	−0.032	0.039	−1.045	0.296	[−0.119, 0.036]
Second year vs. first year	−0.026	−0.009	0.093	−0.278	0.781	[−0.209, 0.157]
Third year vs. first year	−0.324	−0.097	0.077	−4.199	< 0.001	[−0.476, −0.173]
Fourth year vs. first year	−0.020	−0.006	0.091	−0.215	0.830	[−0.198, 0.159]
Science vs. engineering	−0.272	−0.089	0.089	−3.059	0.002	[−0.446, −0.097]
Agriculture vs. engineering	0.047	0.016	0.082	0.567	0.571	[−0.115, 0.208]
Other discipline vs. engineering	−0.799	−0.117	0.129	−6.179	< 0.001	[−1.053, −0.545]
Intolerance of uncertainty	0.286	0.192	0.050	5.717	< 0.001	[0.188, 0.384]
Future time perspective	−0.791	−0.469	0.060	−13.219	< 0.001	[−0.909, −0.674]
Psychological flexibility	−0.146	−0.123	0.037	−3.963	< 0.001	[−0.218, −0.074]
FTP × PF	−0.141	−0.086	0.039	−3.571	< 0.001	[−0.218, −0.063]

Panel D. Model fit and robust block-change tests						
Step	Variables newly entered	*R* ^2^	Adjusted *R*^2^	Δ*R*^2^	HC3 Wald χ^2^(*df*)	*p*
1	Sex, age, year, discipline	0.053	0.044	0.053	67.550 (8)	< 0.001
2	IU, FTP, PF	0.474	0.468	0.421	846.928 (3)	< 0.001
3	FTP × PF	0.481	0.474	0.007	12.750 (1)	< 0.001

*N* = 855. Reference groups were male, first year, and engineering; “Other discipline” pooled the remaining disciplines. Intercepts are omitted. B and β denote the unstandardized and step-specific standardized coefficients, respectively. Each step was estimated separately; HC3 robust SEs, t, p, and CIs refer to B. R^2^, adjusted R^2^, and ΔR^2^ are conventional OLS indices; HC3 Wald χ^2^ tests the robust contribution of each newly entered block, not ΔF. For FTP × PF, β standardizes the constructed product term. For the FTP × PF interaction, Cohen’s f^2^ was calculated from the full-model R^2^ as 0.007/(1–0.481) = 0.013.

**Figure 3:**
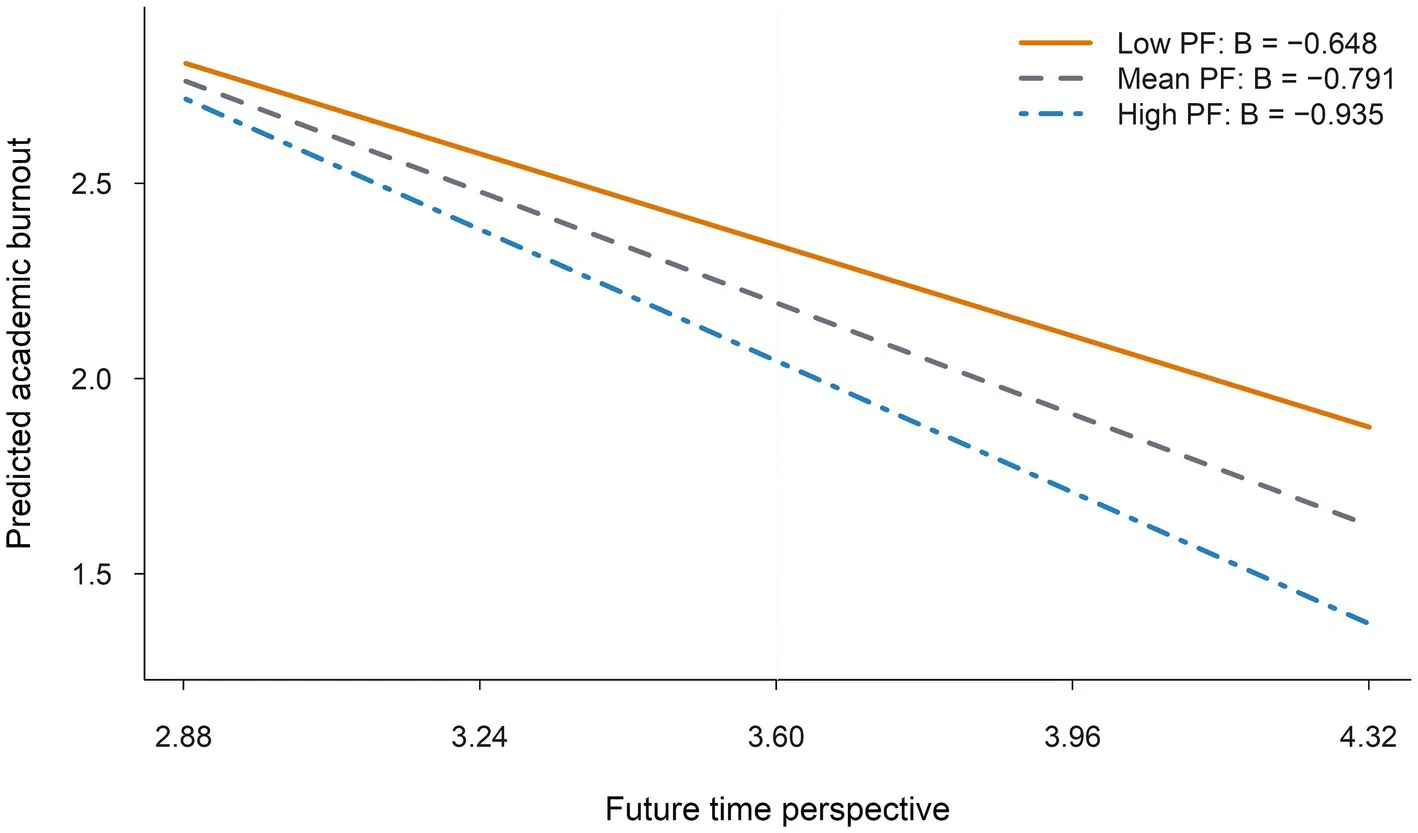
Simple slopes for the association between future time perspective and academic burnout at low, mean, and high levels of psychological flexibility. *N* = 855. Lines show model-implied predicted academic burnout at low (−1 SD), mean, and high (+1 SD) levels of psychological flexibility. Future time perspective and psychological flexibility were mean-centered for estimation, and future time perspective is displayed on its original scale. Intolerance of uncertainty and the prespecified covariates were held at their sample means. The unstandardized simple slopes were *B* = −0.648, −0.791, and −0.935, respectively; all *p* < 0.001.

The results showed that the interaction term between future time perspective and psychological flexibility was significantly associated with academic burnout, *B* = −0.141, HC3 *SE* = 0.039, *t* = −3.571, *p* < 0.001, 95% CI [−0.218, −0.063]. Although statistically significant, the interaction explained only an additional 0.7% of the variance (Δ*R*^2^ = 0.007; Cohen’s *f*^2^ = 0.013), indicating a negligible incremental effect. Simple slope analyses (Figure 3) showed that future time perspective was significantly and negatively associated with academic burnout at low (−1 *SD*; *B* = −0.648, *SE* = 0.080, *p* < 0.001, 95% CI [−0.806, −0.490]), mean (*B* = −0.791, *SE* = 0.060, *p* < 0.001, 95% CI [−0.909, −0.674]), and high levels of psychological flexibility (+1 *SD*; *B* = −0.935, *SE* = 0.063, *p* < 0.001, 95% CI [−1.058, −0.812]). The negative relationship became stronger as psychological flexibility increased. Thus, H3 received statistical support.

Analysis of the conditional indirect effects further showed that the conditional indirect effect of intolerance of uncertainty on academic burnout through future time perspective was statistically significant at low, mean, and high levels of psychological flexibility. Specifically, the indirect effect was 0.236 at a low level of psychological flexibility (Boot*SE* = 0.036, 95% CI [0.169, 0.309]), 0.288 at the mean level (Boot*SE* = 0.033, 95% CI [0.226, 0.356]), and 0.340 at a high level (Boot*SE* = 0.036, 95% CI [0.273, 0.416]). In addition, the index of moderated mediation was statistically significant, index = 0.051, Boot*SE* = 0.015, 95% CI [0.023, 0.082]. Because its bootstrap confidence interval did not include 0, the indirect effect through future time perspective varied across levels of psychological flexibility. Specifically, the indirect effect became stronger as psychological flexibility increased.

### 3.6 Measurement model evaluation and latent-variable supplementary analyses

To evaluate the psychometric basis for using an overall FTP score in the primary analysis, we fitted an FTPS-AYA bifactor model. The model showed good overall fit, scaled χ^2^/*df* = 2.816, scaled RMSEA = 0.046, 90% CI [0.042, 0.050], scaled CFI = 0.987, scaled TLI = 0.984, and SRMR = 0.022, with no evident improper parameter estimates. The general factor showed high omega hierarchical reliability (ωH = 0.906), accounted for 71.0% of the common variance (ECV = 0.710), and had a median item-level explained common variance of 0.765 (median IECV = 0.765). Together, the model-fit results and bifactor indices indicated a strong general FTP factor that adequately summarized the common variance among the items, providing a psychometric basis for using the overall FTP score in the primary PROCESS Model 14 analysis (Table 5).

**Table 5:** Measurement-model fit and FTPS-AYA bifactor summary indices.

Model	Scaled χ^2^/*df*	CFI	TLI	RMSEA (90% CI)	SRMR	Additional reported indices
FTPS-AYA bifactor model	2.816	0.987	0.984	0.046 [0.042, 0.050]	0.022	ωH = 0.906; ECV = 0.710; median IECV = 0.765
Four higher-order-factor substantive model	2.635	0.951	0.950	0.044 [0.042, 0.045]	0.063	CR/AVE: IU = 0.901/0.821; FTP = 0.940/0.724; PF = 0.936/0.711; academic burnout = 0.873/0.698. Maximum |φ| = 0.731 (φ = −0.731, 95% CI [−0.764, −0.698]); maximum HTMT₂ = 0.717, bootstrap 95% CI [0.665, 0.760]
Three-construct model: FTP and PF combined	4.031	0.910	0.907	0.060 [0.058, 0.061]	0.083	χ^2^(2131) = 8589.156; IFI = 0.910
Three-construct model: FTP and academic burnout combined	3.141	0.936	0.934	0.050 [0.049, 0.051]	0.070	χ^2^(2131) = 6694.055; IFI = 0.936
Three-construct model: PF and academic burnout combined	4.091	0.908	0.905	0.060 [0.059, 0.061]	0.085	χ^2^(2131) = 8718.916; IFI = 0.908
67-item single-factor model	9.200	0.755	0.747	0.098 [0.097, 0.099]	0.125	—

All models were estimated with WLSMV. ωH, ECV, and IECV denote omega hierarchical, explained common variance, and item explained common variance, respectively. In three-construct models, only the named pair was combined. Merged-model comparisons are descriptive; no WLSMV χ^2^ difference test was performed. A dash indicates that no additional indices were reported.

Next, we specified a joint higher-order measurement model comprising IU, FTP, PF, and academic burnout. The model showed acceptable fit, scaled χ^2^/*df* = 2.635, scaled CFI = 0.951, scaled TLI = 0.950, scaled RMSEA = 0.044, 90% CI [0.042, 0.045], and SRMR = 0.063. The model showed substantially better fit than the 67-item single-factor model reported in Table 5. Composite reliability and average variance extracted (AVE) met their respective criteria, while the Fornell–Larcker criterion and HTMT₂ values supported discriminant validity. Collectively, these results supported the reliability, convergent validity, and empirical distinctiveness of the four constructs and provided a measurement basis for the subsequent latent-variable supplementary analysis (Table 5).

On this basis, we fitted a strict, covariate-adjusted MLR product-indicator latent interaction model using the same structural paths and covariate specification as the primary observed-score PROCESS Model 14 to examine whether the focal interaction was also evident after accounting for measurement error in the overall constructs. The latent FTP × PF interaction was negative and statistically significant, *B* = −0.173, robust *SE* = 0.072, *p* = 0.016, 95% CI [−0.313, −0.032]. The latent-variable index of moderated mediation was 0.105, *SE* = 0.043, *p* = 0.015, 95% CI [0.021, 0.189]. These results were consistent in direction with the primary analysis based on the scale composite scores and provided supplementary evidence for the second-stage moderation specified in Model 14 (Table 6).

**Table 6:** Observed-score and latent-variable moderation and moderated mediation results.

Analysis/model	Parameter	Estimate	*SE*	*p*	95% CI
Observed-score PROCESS Model 14	FTP × PF interaction	*B* = −0.141	0.039	< 0.001	[−0.218, −0.063]
Strict latent-variable Model 14	FTP × PF interaction	*B* = −0.173	0.072	0.016	[−0.313, −0.032]
Observed-score PROCESS Model 7	IU × PF interaction	*B* = 0.023	0.031	0.461	[−0.037, 0.083]
Observed-score PROCESS Model 5	IU × PF interaction	*B* = 0.022	0.038	0.562	[−0.053, 0.097]
Observed-score PROCESS Model 14	Index of moderated mediation	0.051	0.015	—	[0.023, 0.082]
Strict latent-variable Model 14	Index of moderated mediation	0.105	0.043	0.015	[0.021, 0.189]

*N* = 855. All models controlled for sex, age, year of study, and academic discipline. The same covariates were included in both the FTP and academic-burnout equations. The strict latent-variable Model 14 used the same structural and covariate specification as the primary observed-score PROCESS Model 14. Observed-score interactions used HC3 robust SEs; the observed-score index used a bootstrap SE and a 5,000-resample percentile CI. The strict latent-variable Model 14 used 12 prespecified matched FTP–PF product indicators (mean-centered constituents, double-mean-centered products, no residual centering; std.lv = TRUE). All estimates are unstandardized. Latent inference used MLR robust SEs and Wald CIs; its index used the delta method. A dash denotes inference from the bootstrap CI without a *p* value. FTP, future time perspective; PF, psychological flexibility; IU, intolerance of uncertainty.

PROCESS Model 14 based on the scale composite scores remained the primary model, while Models 7 and 5 were fitted separately to examine alternative moderation placements. In Model 7, the IU × PF interaction was not statistically significant, *B* = 0.023, HC3 robust *SE* = 0.031, *p* = 0.461, 95% CI [−0.037, 0.083]. Similarly, the IU × PF interaction in Model 5 was not statistically significant, *B* = 0.022, HC3 robust *SE* = 0.038, *p* = 0.562, 95% CI [−0.053, 0.097]. Among the three moderation placements examined, statistical support was obtained only for the second-stage interaction specified in Model 14. Overall, the supplementary analyses provided complementary support for the measurement basis of the composite scores and the focal second-stage interaction in Model 14. However, because the data were cross-sectional, these results do not establish temporal ordering or causal mechanisms.

## 4 Discussion

The present study examined the association between intolerance of uncertainty and academic burnout among university students, together with the distinct roles of future time perspective and psychological flexibility in this association. The findings showed that intolerance of uncertainty was positively associated with academic burnout; future time perspective partially mediated this association; and psychological flexibility moderated the association between future time perspective and academic burnout, such that the corresponding indirect association varied across levels of psychological flexibility. Together, these findings bring students’ cognitive appraisal of uncertainty, their understanding of future goals, and goal-directed action under stress into a coherent interpretive framework for understanding academic burnout among Chinese university students.

### 4.1 Relationship between intolerance of uncertainty and academic burnout

The present study found a significant positive association between intolerance of uncertainty and academic burnout among university students. The direct association between intolerance of uncertainty and academic burnout remained after adjustment for future time perspective, psychological flexibility, their interaction, and the covariates. This finding is generally consistent with previous research. Qiang et al. (2024) reported a positive association between intolerance of uncertainty and academic burnout among university students, with self-regulatory fatigue playing a mediating role. Lin and Huang (2014) showed that life stress among university students, particularly academic stress and stress related to future development, was closely associated with higher academic burnout. Zuo et al. (2024) further found that academic stress was linked to academic burnout through rumination. Together, these studies suggest that academic burnout is related not only to external academic demands and the level of stress but also to how students appraise stress, process negative information, and respond to uncertainty.

Within the context of Chinese higher education, examination outcomes, grade rankings, decisions about postgraduate study, and employment prospects represent potential sources of uncertainty for university students. Although the present study did not directly measure these specific circumstances, they provide a contextual basis for interpreting the observed associations. Students with higher intolerance of uncertainty may appraise these circumstances not only as ordinary academic challenges but also as potential threats. They may repeatedly question whether the future is controllable, whether their efforts will yield the expected returns, and whether failure will have serious consequences. As these appraisals accumulate, existing academic pressure may be amplified and become associated with persistent worry, tension, and psychological burden.

From a theoretical perspective, appraising uncertain situations as threatening is an important manifestation of intolerance of uncertainty. Intolerance of uncertainty reflects negative cognitive, emotional, and behavioral responses to ambiguous situations (Freeston et al., 1994). Previous research has shown that individuals with higher intolerance of uncertainty tend to view uncertain outcomes as unacceptable even when adverse events are relatively unlikely to occur (Carleton et al., 2007). In academic contexts, this tendency may be expressed as excessive attention to possible examination failure, academic underperformance, obstacles to postgraduate study, or employment difficulties. Whether students remain chronically preoccupied with anticipating, monitoring, and worrying about potential risks, rather than the objective presence of these risks alone, may help explain the association between intolerance of uncertainty and academic burnout.

From the perspectives of conservation of resources theory and the demands–resources framework, one possible theoretical explanation is that uncertainty regarding academic and future development may constitute a demand that requires the continued investment of psychological resources for regulation (Hobfoll, 1989; Demerouti et al., 2001). Students with higher intolerance of uncertainty may need to devote more cognitive and emotional resources to predicting, monitoring, or controlling outcomes that have not yet been determined. From this theoretical perspective, they may also be more inclined to seek certainty repeatedly or to avoid academic tasks with ambiguous outcomes or high levels of difficulty. These possible coping responses may temporarily alleviate distress, but they may not change actual academic demands or strengthen students’ sense of control over learning. Under this interpretation, persistent worry, resource expenditure, and avoidance may be associated with lower academic engagement, emotional exhaustion, detachment from learning, and reduced academic efficacy.

### 4.2 Mediating role of future time perspective

The findings supported a significant partial mediating role of future time perspective in the association between intolerance of uncertainty and academic burnout. Students with higher intolerance of uncertainty tended to report lower future time perspective, which was in turn associated with higher academic burnout. This pattern suggests that understanding the association between intolerance of uncertainty and academic burnout requires attention not only to students’ worry and expenditure of psychological resources when facing uncertainty, but also to how they understand the future and whether they can connect their current learning with future goals. Qiang et al. (2024) explained this association from the perspective of self-regulatory fatigue, whereas the present study supplements this account by focusing on future-oriented cognition and goal construction. These two pathways concern the expenditure of psychological resources and the organization of future goals, respectively, and suggest that different psychological processes may underlie the association between intolerance of uncertainty and academic burnout.

First, intolerance of uncertainty may be associated with lower future time perspective. Future development inherently involves many unknowns, and postgraduate study, employment, professional development, and personal growth cannot be predicted with complete certainty. Students with higher intolerance of uncertainty may focus more on the possibility of developmental setbacks, efforts failing to yield expected outcomes, and events beyond their control, while devoting less attention to setting goals, planning pathways, and continuing to act toward those goals. This threat-focused view of the future may be associated with a weaker sense of future clarity, perceived control, and goal orientation. Yang et al. (2021) reported a negative association between intolerance of uncertainty and future time perspective, which is consistent with the direction observed in the present study.

Second, future time perspective is related to how students understand the long-term value of their current learning. Students with clearer future goals are more likely to view coursework, examinations, research training, and skill development as necessary steps toward their future goals, thereby giving their current learning clearer meaning and direction and sustaining goal persistence and academic engagement (Husman and Shell, 2008; De Bilde et al., 2011). In contrast, when students lack a clear future direction, their current learning may be experienced as repetitive, burdensome, or meaningless and may be associated with reduced academic motivation, emotional exhaustion, and doubts about the value of learning. Research with Chinese undergraduates has also shown that future time perspective is negatively associated with academic burnout and its dimensions (Hong and Hanafi, 2024). Future time perspective has also been reported to mediate the association between mindfulness and learning burnout among university students (Yang et al., 2023). These findings are consistent with those of the present study.

Taken together, future time perspective may connect students’ understanding of uncertainty with their appraisal of the meaning of their current learning. Students with higher intolerance of uncertainty may be more likely to view the future in threat-focused terms, and this cognitive tendency may be associated with a weaker sense of goals, direction, and control. When future goals become less clear, students may find it more difficult to place their current learning within a broader framework of long-term development, and their understanding of the value of learning and sustained engagement may consequently weaken. Thus, the future-oriented cognition, goal organization, and perceived meaning of learning reflected in future time perspective provide one possible theoretical explanation for the indirect association between intolerance of uncertainty and academic burnout.

### 4.3 Moderating role of psychological flexibility

The present study found that psychological flexibility moderated the association between future time perspective and academic burnout. The indirect association between intolerance of uncertainty and academic burnout through future time perspective also varied across levels of psychological flexibility. Specifically, future time perspective was associated with lower academic burnout at different levels of psychological flexibility, and this negative association was more pronounced among students with higher psychological flexibility. However, although the moderating effect was statistically supported, the interaction explained only an additional 0.7% of the variance (Δ*R*^2^ = 0.007; Cohen’s *f*^2^ = 0.013), indicating a negligible incremental effect. This *f*^2^ value falls below Cohen’s (1988) conventional reference value of 0.02 for a small effect. Accordingly, the statistical significance of the interaction should not be interpreted as evidence of substantial practical importance. Psychological flexibility is therefore better understood as part of a statistically detectable conditional pattern with negligible incremental explanatory value, rather than as a strong moderator of academic burnout.

This finding is broadly consistent with the core meaning of psychological flexibility. Psychological flexibility emphasizes remaining open to and aware of present experiences while continuing to act in accordance with personal goals and values in the presence of stress, anxiety, and discomfort (Hayes et al., 2006; Kashdan and Rottenberg, 2010). Previous studies have also linked psychological flexibility to adaptive action, effective coping, and engagement in meaningful activities under challenging and stressful conditions (Bond et al., 2011; Levin et al., 2012; Gloster et al., 2017). The moderation observed in the present study is consistent with applying this theoretical perspective to the context of academic burnout, suggesting that psychological flexibility may be relevant to the association between future goals and lower academic burnout when students continue to act in accordance with their goals while experiencing discomfort.

From the perspective of the learning process, future time perspective may help students connect current tasks with long-term goals, thereby giving learning activities clearer meaning and direction (Zimbardo and Boyd, 1999; Husman and Shell, 2008; Lyu and Huang, 2016). However, having future goals does not necessarily mean that students can sustain goal-directed action under real-world stress. University students continue to face course demands, examination pressure, experiences of failure, and uncertainty about future development while pursuing their goals. Theoretically, students with lower psychological flexibility may be more likely to engage in avoidance, become disengaged, or reduce their academic effort even when they have future goals. Students with higher psychological flexibility may be more inclined to adjust their learning behavior and sustain goal-consistent behavior. Accordingly, the association between future time perspective and lower academic burnout was more pronounced among students with higher psychological flexibility.

Overall, psychological flexibility can theoretically be understood as an adaptive capacity related to goal-directed action. It does not directly eliminate academic stress or uncertainty, but is related to whether students can continue adjusting their actions in accordance with future goals while experiencing stress and negative feelings. Among students with higher psychological flexibility, the association between future time perspective and academic burnout was more pronounced. This finding is consistent with the interpretation that psychological flexibility may shape the strength of the association between future goals and academic burnout. In addition, neither Model 5 nor Model 7 clearly supported the two alternative moderation placements, providing relative support for specifying psychological flexibility as a moderator of the second-stage path.

## 5 Contributions and implications

At the theoretical level, the main contribution of this study lies in integrating uncertainty appraisal, future-oriented cognition, and goal-consistent action under stress within a single conditional process framework. This moves beyond examining correlates of academic burnout separately to consider how uncertainty appraisal may be related to academic burnout and under what conditions this association varies. Previous research has largely explained academic burnout in terms of academic stress, workload, social support, learning motivation, and individual psychological characteristics, focusing mainly on the demands students face and the coping resources available to them (Schaufeli et al., 2002; Walburg, 2014; Zuo et al., 2024). Previous research has also found that intolerance of uncertainty is associated with academic burnout and, drawing on limited-resource theory, has proposed resource depletion reflected in self-regulatory fatigue as one pathway connecting the two (Qiang et al., 2024). Building on this work, the present study examined whether intolerance of uncertainty was associated with lower future time perspective and whether the association between future time perspective and academic burnout varied across levels of psychological flexibility.

The observed pattern was consistent with this theoretical account: higher intolerance of uncertainty was associated with a lower overall future time perspective score, which was in turn associated with higher academic burnout, and the association between future time perspective and academic burnout varied across levels of psychological flexibility. Future time perspective therefore reflects whether students can understand the meaning of current learning from a long-term developmental perspective and form clear future goals and direction, whereas psychological flexibility reflects whether students can continue to act in accordance with future goals when anxiety, uncertainty, and setbacks are present. Psychological flexibility was placed in the second stage to test whether it was associated with variation in the strength of the future time perspective–academic burnout association rather than treating it as an independent correlate considered alongside academic burnout. Among the separately estimated moderation models, only the interaction specified in Model 14 received statistical support; however, its incremental explanatory value was negligible. Overall, this study brought uncertainty appraisal, future-oriented cognition, and goal-consistent action under stress into the same explanatory framework to clarify the indirect association between intolerance of uncertainty and academic burnout through future time perspective and how this indirect association varied across levels of psychological flexibility. The results therefore provide statistically detectable but limited support for the proposed conditional pattern.

In practical terms, the present cross-sectional findings identify questions for future research rather than establishing an intervention framework. The associations involving intolerance of uncertainty and future time perspective may help generate hypotheses about whether approaches addressing uncertainty appraisal or future-oriented goal setting could be evaluated among university students. However, the negligible incremental effect of the FTP × PF interaction does not support prioritizing psychological flexibility as an intervention target or tailoring student support according to students’ psychological-flexibility levels. The moderation result should therefore be treated as hypothesis-generating. Longitudinal and experimental studies are needed to determine whether changing future time perspective or psychological flexibility produces meaningful reductions in academic burnout.

## 6 Limitations and future research

This study has several limitations. First, it used a cross-sectional design, which can only indicate associations among intolerance of uncertainty, future time perspective, psychological flexibility, and academic burnout. It cannot establish their temporal ordering or causal relationships. The observed associations may also be compatible with reverse or reciprocal relationships. For example, prolonged academic burnout may weaken students’ future orientation or psychological flexibility. Longitudinal studies are needed to examine temporal ordering and within-person change. Well-controlled intervention studies could separately evaluate whether programs targeting future time perspective or psychological flexibility reduce academic burnout.

Second, the study relied exclusively on self-report questionnaire data. Although the preliminary CMV assessments did not support a dominant single-factor explanation, common method bias cannot be ruled out. Students’ responses may still have been affected by social desirability, individual differences in item interpretation, current emotional state, and response habits. The concurrent use of conceptually related self-report measures also means that shared method variance and construct overlap may have contributed to the observed associations. Future research could combine self-reports with academic achievement, course participation records, teacher or peer evaluations, and interviews. Relevant covariates, including objective or self-reported academic performance, could also be incorporated when theoretically justified.

Third, convenience sampling and uneven representation across regions, university types, majors, and grade levels limit the generalizability of the findings. Students across these contexts may experience different forms of academic pressure and future-related uncertainty. Future research should recruit more diverse and representative samples and conduct multisite replication studies to assess the model’s robustness across educational contexts.

Fourth, future time perspective was operationalized using the overall score from a multidimensional scale. Although the supplementary latent-variable analysis accounted for measurement error in the overall constructs, it did not identify which FTPS-AYA dimensions accounted for the indirect association. Future research could compare dimension-specific and latent-variable models to clarify how different facets of future time perspective contribute to the observed pattern.

Finally, academic burnout is shaped by multiple psychological and contextual factors. The present study focused on future time perspective and psychological flexibility in relation to intolerance of uncertainty and academic burnout. Future research could examine theoretically specified alternative models incorporating academic self-efficacy, psychological resilience, social support, perceived stress, or career anxiety. Comparing their incremental and combined contributions may provide a more differentiated account of academic burnout.

## 7 Conclusion

This study examined the associations among intolerance of uncertainty, future time perspective, psychological flexibility, and academic burnout in a cross-sectional sample of Chinese university students. At the theoretical level, the study integrated uncertainty appraisal, future-oriented cognition, and goal-consistent action under stress within a single framework to examine the indirect association between intolerance of uncertainty and academic burnout and how it varied across levels of psychological flexibility. Higher intolerance of uncertainty was associated with both higher academic burnout and lower future time perspective, while lower future time perspective was associated with higher academic burnout; the direct association between intolerance of uncertainty and academic burnout also remained. The negative association between future time perspective and academic burnout was more pronounced at higher levels of psychological flexibility, although the interaction had a negligible incremental effect (Δ*R*^2^ = 0.007; Cohen’s *f*^2^ = 0.013). Consistent with this pattern, the conditional indirect effect of intolerance of uncertainty on academic burnout through future time perspective was stronger at higher levels of psychological flexibility. These findings may help generate hypotheses for future longitudinal and experimental research on uncertainty appraisal, future-oriented cognition, psychological flexibility, and academic burnout, but they do not establish that interventions targeting these constructs would reduce academic burnout.

## Data Availability

The de-identified data supporting the conclusions of this article will be made available by the corresponding author upon reasonable request, subject to applicable ethical and privacy requirements.
